# Somatic Experiencing for Posttraumatic Stress Disorder: A Randomized Controlled Outcome Study

**DOI:** 10.1002/jts.22189

**Published:** 2017-06-06

**Authors:** Danny Brom, Yaffa Stokar, Cathy Lawi, Vered Nuriel‐Porat, Yuval Ziv, Karen Lerner, Gina Ross

**Affiliations:** ^1^ Herzog Israel Center for the Treatment of Psychotrauma Jerusalem Israel; ^2^ Paul Baerwald School of Social Work and Social Welfare the Hebrew University of Jerusalem Jerusalem Israel; ^3^ International Trauma‐Healing Institute Los Angeles, California USA/Ra'anana Israel; ^4^ Tel Aviv Israel

## Abstract

This study presents the first known randomized controlled study evaluating the effectiveness of somatic experiencing (SE), an integrative body‐focused therapy for treating people with posttraumatic stress disorder (PTSD). There were 63 participants meeting *DSM‐IV‐TR* full criteria for PTSD included. Baseline clinical interviews and self‐report measures were completed by all participants, who were then randomly assigned to study (*n* = 33) or waitlist (*n* = 30) groups. Study participants began 15 weekly SE sessions, whereas waitlist participants waited the same period, after which the second evaluation was conducted. All participants were evaluated a third time after an additional 15 weeks, during which time the waitlist group received SE therapy. Pretreatment evaluation showed no significant differences between groups. Mixed model linear regression analysis showed significant intervention effects for posttraumatic symptoms severity (Cohen's *d* = 0.94 to 1.26) and depression (Cohen's *d* = 0.7 to 1.08) both pre‐post and pre‐follow‐up. This randomized controlled study of SE shows positive results indicating SE may be an effective therapy method for PTSD. Further research is needed to understand who shall benefit most from this treatment modality.

## VIVENCIAS SOMÁTICAS PARA EL TRASTORNO DE ESTRÉS POS‐ TRAUMÁTICO: UN ESTUDIO CONTROLADO RANDOMIZADO

Resumen

VIVENCIAS SOMATICAS PARA TEPT

Este estudio presenta el primer estudio controlado randomizado conocido para evaluar la efectividad de las Vivencias Somáticas (VS), una terapia integrativa enfocada en el cuerpo para tratar a las personas con trastorno de estrés pos traumático TEPT). Sesenta y tres participantes que cumplieron con el criterio completo del DSM IV‐TR (4ª ed., text.rev.; DSM‐IV‐TR; Sociedad Americana de Psiquiatría, 2000) para TEPT fueron incluidos. Entrevistas clínicas de línea base y medidas de auto‐reporte fueron completados por todos los participantes, quienes fueron asignados azarosamente al grupo estudio (n = 33) o lista de espera (n = 30). Los participantes del grupo estudio comenzaron 15 sesiones semanales de VS, mientras que los participantes de la lista de espera esperaron el mismo periodo de tiempo, después del cual la segunda evaluación fue conducida. Todos los participantes fueron evaluados una tercera vez después de 15 semanas adicionales, durante este tiempo, el grupo de lista de espera recibió terapia VS. La evaluación de pretratamiento no mostró diferencias significativas entre los grupos. Análisis de regresión lineal de modelo mixto mostró efectos significativos de intervención para la severidad de los síntomas postraumático (Cohen's d = 0.94 a 1.26) y depresión (Cohen's d = 0.7 a 1.08) ambos pre–post y pre‐seguimiento. Este estudio controlado randomizado de VS muestra resultados positivos indicando que VS puede ser un método terapéutico efectivo para TEPT. Se necesita más investigación para entender quiénes pueden beneficiarse más de esta modalidad de tratamiento.

Estudios prospectivos describen y predicen diferencias individuales en el curso de los síntomas de estrés postraumático (SEPT) después de los desastres son escasos. El presente estudio tiene como objetivo describir y predecir las diferencias individuales en el nively la velocidad de cambio en SEPT después de la bomba de Oslo en 2011, que fue un ataque terrorista dirigido al gobierno Noruego. Los dato de encuestas de empleados ministeriales (N = 256) fueron recogidos 10, 22 y 34 meses después del bombardeo. Utilizamos el modelo de crecimiento latente para examinar el desarrollo del SEPT, y para identificar la fuerza de lasvariables predictorespotenciales. Alta exposición, siendo mujer, y altos niveles de neuroticismo se asociaron con altos niveles de SEPT 10 meses después del evento traumático (β vario de .25 a .30, *p* < .001), mientras el apoyo social se asoció con menores niveles de SEPT (β = –.30, *p* < .001).Ser mujer con alto neuroticismo se asoció con una rápida reducción de SEPT. (βvario de–.20 to .39, *p* < .05).Una alta exposición parece tener una influencia duradera mediante la mantención de altos niveles de SEPT. Nuestros hallazgos sugieren que ser mujer, ser altamente expuesta, y tener bajos niveles de apoyo social fueron marcadores de riesgo para el SEPT duradero.

## Somatic Experiencing for Post‐Traumatic Stress Disorder: A Randomized Controlled Outcome Study

Traditional Chinese

標題: 為治療創傷後壓力症的體感治療༚一項隨機對照的臨床實效研究

撮要: 這是首個隨機對照研究, 檢視為創傷後壓力症(PTSD)患者提供、針對軀體的綜合療法──體感治療(SE)的功效。樣本包含63名完全符合DSM IV‐TR (精神疾病診斷與統計手冊第四修訂版; 美國精神醫學學會, 2000) PTSD準則的人士。所有參與者均完成了基線的臨床面談和自評測量。他們隨後被隨機分派到研究組(n = 33)和等待組(n = 30)。研究組進行每週1次、共15次的SE節段, 其後我們對等待組和研究組進行第二次評估。然後, 等待組接受SE, 我們在15週後再為兩組進行第三次評估。在療法前的評估結果裡, 兩組之間並無顯著差異。然而, 線性迴歸混合模型分析反映, 在治療前後(pre‐post)及治療前與跟進後(pre‐follow‐up), 創傷後症狀嚴重度(Cohen's d = 0.94 至 1.26)和抑鬱症(Cohen's d = 0.7 至 1.08)有顯著的干預效應。這個對SE的隨機對照研究反映正向結果, 顯示SE可能是對PTSD患者有效的療法。未來需有更多研究以找出此治療方式對哪種人最為有效。

Simplified Chinese

标题: 为治疗创伤后压力症的体感治疗༚一项随机对照的临床实效研究

撮要: 这是首个随机对照研究, 检视为创伤后压力症(PTSD)患者提供、针对躯体的综合疗法──体感治疗(SE)的功效。样本包含63名完全符合DSM IV‐TR (精神疾病诊断与统计手册第四修订版; 美国精神医学学会, 2000) PTSD准则的人士。所有参与者均完成了基线的临床面谈和自评测量。他们随后被随机分派到研究组(n = 33)和等待组(n = 30)。研究组进行每周1次、共15次的SE节段, 其后我们对等待组和研究组进行第二次评估。然后, 等待组接受SE, 我们在15周后再为两组进行第三次评估。在疗法前的评估结果里, 两组之间并无显著差异。然而, 线性回归混合模型分析反映, 在治疗前后(pre‐post)及治疗前与跟进后(pre‐follow‐up), 创伤后症状严重度(Cohen's d = 0.94 至 1.26)和抑郁症(Cohen's d = 0.7 至 1.08)有显著的干预效应。这个对SE的随机对照研究反映正向结果, 显示SE可能是对PTSD患者有效的疗法。未来需有更多研究以找出此治疗方式对哪种人最为有效。

The treatment of posttraumatic stress disorder (PTSD) has been the topic of much research. Even though effective methods have been established (Benish, Imel, & Wampold, 2008; Cusack et al., 2016; Foa, Keane, Friedman, & Cohen, 2008; Haagen, Smid, Knipscheer, & Kleber, 2015), no method has been shown to work for all people suffering from PTSD. Besides evidence‐based treatment methods such as those based on cognitive–behavioral theory, including prolonged exposure (Foa et al., 2008), cognitive processing therapy (Resick, & Schnicke, 1992), brief eclectic psychotherapy (Gersons, Carlier, Lamberts, & van der Kolk, 2000), and eye movement desensitization and reprocessing (Shapiro, 1989), quite a number of additional methods have been proposed, but have not yet been studied extensively. We studied a 15‐session protocol of somatic experiencing (SE; Levine, 2010) in a randomized controlled trial to assess its effectiveness vis‐a‐vis a waitlist control group.

SE (Levine, 2010) is a body‐focused therapy used for treating people suffering from PTSD that integrates body awareness into the psychotherapeutic process, taking a unique approach not used by other PTSD treatment methods. The focus of the therapy is on creating awareness of inner physical sensations, which are seen as the carriers of the traumatic memory. In the theory behind SE (Levine, 2010), posttraumatic stress symptoms are considered an expression of stress activation and an incomplete defensive reaction to a traumatic event. From this theoretical perspective, the goal of the therapy is to release the traumatic activation through an increased tolerance of bodily sensations and related emotions, inviting a discharge process to let the activation dissipate. SE differs from exposure therapy methods used for treating PTSD in that it does not require extensive nor full retelling of the traumatic events. It does require the client to engage with traumatic memories that cause high arousal. The client learns to monitor the arousal and downregulate it in an early phase by using body awareness, and applying self‐regulatory mechanisms like engagement in pleasant sensations, positive memories, or other experiences that help regulate arousal. The therapeutic goal is to decrease the distress and symptoms caused by the posttraumatic arousal and restore healthy functioning in daily life (Levine, 2010; Payne, Levine, & Crane‐Godreau, 2015).

To date, the literature on the effectiveness of SE is scarce and scientifically insufficient. Parker, Doctor, and Selvam (2008) offered a single 75‐minute session to 204 survivors of the 2004 tsunami in southern India. Out of the 150 participants who completed the follow‐up assessments 4 and 8 months later, 90% of the participants reported significant improvement or being completely free of symptoms of intrusion, arousal, and avoidance, based on the Impact of Events Scale (Horowitz, Wilner, & Alvarez, 1979). Acknowledging the lack of a control group, Parker et al. state their belief that SE helped resolve posttraumatic symptoms.

An additional post‐Tsunami intervention described by Leitch (2007) included 53 participants, aged 3 to 75 years, who received one to two sessions of treatment 1‐month post‐tsunami, with a repeated evaluation 1 year later. Results, based on a symptom tracking form developed by the research team, demonstrated that immediately after the SE session 67.0% showed complete or partial improvement in reported symptoms. One year later, 90.0% of the 22 participants located reported maintaining this improvement. Here, too, the author acknowledges the exploratory nature of the study, and called for caution interpreting results based on the convenience sample, the lack of a comparison group, and the small sample size at follow‐up.

Although other methods have become common practice after clinical trials proved their effectiveness (Foa et al., 2008), the effectiveness of SE had not yet been proven in a randomized controlled setting; therefore, the aim of this study was to examine SE in a randomized controlled study.

## Method

### Participants

Over the course of 3 years, 63 participants meeting eligibility criteria were included in the study, 32 women (50.7%) and 31 men (49.2%). Participants were over the age of 18 years (*M* = 40.51, *SD* = 13.05), fluent in either Hebrew or English, and all participants met the *DSM ‐IV‐TR* criteria for full PTSD resulting from one or more single traumatic events. Once they completed a full assessment, participants were randomly assigned to one of two groups: the study group *(n* = 33) or the waitlist control group (*n* = 30). Pretreatment evaluation showed a significant difference between the two groups regarding age (intervention group: *M* = 37.2 years, *SD* = 12.7; waitlist group: *M* = 44.5, *SD* = 12.7, *t* = 2.26, degrees of freedom [*df* ] = 61, *p* = .027), but no significant differences regarding other sociodemographic characteristics (including gender, marital status, education, employment, and religious affiliation, see Table 1). There was also no significant difference in the number of years that elapsed since the traumatic event (intervention group: *M* = 3.9, *SD* = 5.8; waitlist group: *M* = 4.2, *SD* = 6.7, *t* = 0.23, *df* = 60, *p* = .822).

**Table 1:** Demographic Characteristics of the Sample Pooled and by Group

Variable	Total (*n* = 63)		Intervention (*n* = 33)		Control (*n* = 30)		Comparison Tests		
	*N*	%	*n*	%	*n*	%	*df*	*χ* ^2^	*p*
Gender							1	0.79	.374
Female	32	50.8	15	45.5	17	56.7			
Male	31	49.2	18	54.5	13	43.3			
Marital status							4	2.47	.649
Married	38	60.3	22	66.7	16	53.3			
Single	16	25.4	8	24.2	8	26.7			
Divorced	6	9.5	2	6.1	4	13.3			
Widowed	1	1.6	0	0.0	1	3.3			
Other	2	3.2	1	3.0	1	3.3			
Education							5	7.03	.218
Academic	25	39.7	10	30.3	15	50.0			
Partial matriculation	11	17.5	6	18.2	5	16.7			
Full matriculation	7	11.1	4	12.1	3	10.0			
≥12 years	13	20.6	9	27.3	4	13.3			
Vocational	4	6.3	1	3.0	3	10.0			
Religious study	3	4.8	3	9.1	0	0.0			
Employment status							2	0.78	.677
Salaried worker	39	61.9	20	60.6	19	63.3			
Unemployed	13	20.6	6	18.2	7	23.3			
Self‐employed	11	17.5	7	21.2	4	13.3			
Religious affiliation							3	4.14	.247
Secular	25	39.7	10	30.3	15	50.0			
Traditional	18	28.6	11	33.3	7	23.3			
Modern Orthodox	9	14.3	4	12.1	5	16.6			
Ultra‐Orthodox	11	17.5	8	24.2	3	10.0			

Participants presented with a wide variety of traumatic events triggering PTSD including 28 vehicle accidents (44.4%), 8 assault cases (12.7%), 8 terrorist attacks (12.7%), 7 “other” types of accidents (17.5%), 5 cases of death or injury of a family member (7.9%), 4 cases of medical trauma (6.3%), 2 combat cases (3.2%), and 1 threat case (1.6%). No significant differences were found in group allocation.

### Procedure

The study was conducted in Israel by the Herzog Israel Center for the Treatment of Psychotrauma (ICTP) together with the International Trauma‐Healing Institute (ITI). The study, including procedure, expected outcomes, benefits, and potential risks, was presented to Herzog Hospital's Institutional Review Board (Jerusalem, Israel) by the principal investigator and research coordinator, and received the board's written approval. In the time period during which the study took place, several highly stressful national events also occurred. In both 2012 and 2014, wars took place between Israel and the Palestinian factions in Gaza. In addition, there were many terrorist attacks in the Jerusalem area, and all the participants were exposed to this directly or indirectly. We have not included the measurement of this exposure in this study.

The participants were referred to ICTP for the purpose of the study via Israeli medical and mental health clinics and practitioners. Short lectures about SE and the study were held during staff meetings at the respective clinics; recruitment brochures were distributed; and ads were placed in local newspapers.

**Figure 1:**
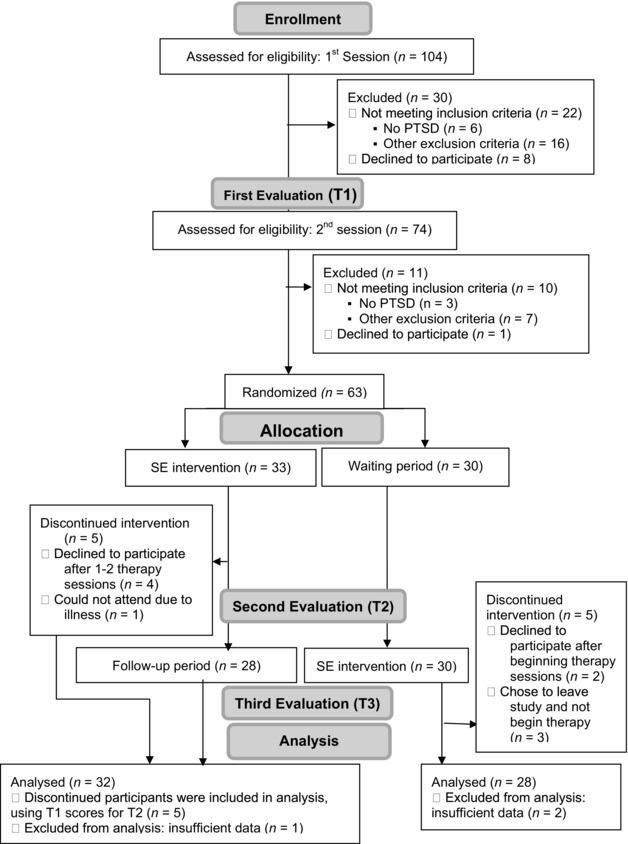
Recruitment and retention flowchart based on CONSORT guidelines (Schulz, Altman, & Moher, 2010). PTSD = posttraumatic stress disorder; SE = somatic experiencing.

Applicants participated in a brief initial phone screening conducted by the research coordinator that consisted of questions about the traumatic events, psychiatric history, and prior traumatic experiences. Applicants who met the initial inclusion criteria were invited for a more extensive clinical assessment (see Figure 1). The clinical assessment (T1), which took place at ICTP, included two parts. In the first session, held with the research coordinator, applicants received a detailed explanation of the study's course, and gave written consent followed by an open interview of the traumatic events and sequelae. At the end of the interview, applicants were asked to complete a set of questionnaires. In the second interview, a trained clinical examiner conducted a clinical interview verifying the presence of PTSD (based on *DSM‐IV‐TR* criteria) using the Clinician‐Administered PTSD Scale (CAPS; Blake et al., 1995).

Applicants were excluded from the study if during the course of the evaluation one of the following conditions arose: a history of psychosis, brain damage, active suicidal tendencies, substance use, psychiatric comorbidity apart from depression, or complex traumatic situations that are characterized by prolonged situations of extreme stress. These were assessed using the Structured Clinical Interview for *DSM‐IV* (SCID; Spitzer, Williams, Gibbon, & First, 1992). Participants who were taking psychiatric medication for over 2 months were included, with the stipulation that any changes made during the course of the study would be made known to the research coordinator. This occurred in two such instances among the waitlist group: one participant stopped taking a selective serotonin reuptake inhibitor antidepressive medication during therapy and a second increased the dosage of an selective serotonin reuptake inhibitor. Applicants who did not meet study criteria were referred by the coordinator back to their health insurance outpatient clinics for therapeutic intervention.

There were 104 applicants enrolled in the study, out of which 30 applicants were excluded after the initial phone screening, and an additional 11 applicants were excluded during the course of the first evaluation. Of those participants who were excluded, 9 did not meet PTSD criteria, 9 decided not to participate in the study, and 23 met an exclusion criterion as listed above, such as active suicidal tendencies, substance use, or psychiatric comorbidity (see Figure 1).

At the conclusion of the evaluation process, 63 applicants who continued to meet inclusion criteria were accepted to the study and assigned by the research coordinator to one of two groups (intervention or waitlist), based on a predetermined list created prior to the beginning of the study (by a flip of a coin, the research coordinator created a list of 100 places, assigning each of them to either "intervention" or "waitlist"). Each participant accepted to the study was assigned the next free spot on the list. The list was only accessed after the participant was accepted, by the research coordinator alone, insuring that all clinical examiners and therapists remained blinded to group allocation, and that the randomization process was not contaminated. Participants assigned to the intervention group began 15 weekly 1‐hr sessions, and participants assigned to the waitlist group waited an equal period of time without any intervention.

At the end of the SE treatment, the intervention group participants met with a clinical examiner for a second assessment (T2) using the same clinical interviews and questionnaires as the initial assessment (T1). Participants assigned to the waitlist group also participated in the second assessment at the end of their 15‐week waiting period, after which they received 15 weekly therapy sessions identical to those in which the participants from the intervention group had taken part. The clinical examiners performing the assessments were trained and supervised by highly experienced trainers in the use of CAPS and SCID, and were blind to the group allocation of the participants. Because both groups were evaluated at the end of a 15‐week period, the examiner could not know the group allocation of the participant, even if he or she had assessed the same participant earlier. Additionally, participants were asked not to talk about their group allocation and whether they had already received therapy. A third and final evaluation (T3) took place 15 weeks after the second evaluation.

During the course of the study, 10 participants (5 from the intervention group and 5 from the waitlist group) did not complete the process (Figure 1). In the intervention group, four participants decided not to seek therapy after one or two SE therapy sessions, and one patient experienced a recurrence of physical illness between T1 and T2. In the waitlist group, three participants chose to leave the study and not begin therapy, and two additional participants left during their therapy sessions. All participants reached the decision on their own accord and were then contacted by the research coordinator. To the best of our knowledge, all decisions were made for various personal reasons (e.g., “too much effort,” “too far”) and not for reasons related to the actual therapy sessions or their content. Again, to the best of our knowledge, there were no adverse reactions reported by the therapists or participants.

For the study, seven therapists with extensive previous experience treating PTSD were recruited. All seven therapists were health care professionals, psychologists, or clinical social workers, licensed by the Israeli Ministry of Health, and were all qualified SE experts (SE practitioners) certified by the Foundation of Human Enrichment (FHE) in the United States. A 15‐session therapy protocol was created for the study, detailing the materials and therapeutic work to be covered in each of the sessions. Therapists were instructed to follow the protocol at hand, and received individual supervision and a number of additional group supervision meetings for all therapists. The supervision ensured therapists adhered to the study protocol. In addition, the therapists were kept blind to participant's group allocation to minimize bias and avoid situations where therapists “try harder” with certain participants, even if on a subconscious level.

The first sessions were dedicated to learning about SE and building therapist–client rapport. The psychoeducational materials covered included basic SE concepts: the concept of trauma, healing through the body, the trauma and healing vortices, experiencing the "felt sense," titration (how to keep arousal at a low level during the processing of traumatic triggers), pendulation (balancing between regulated parts in the body and dysregulated parts), and discharge (how to make arousal dissipate). Therapeutic work began by teaching participants how to regulate the body through identifying and/or creating a list of resources to be used to reduce arousal. Once a sense of stability was created, advanced SE concepts were discussed like tracking the sensations, images, behavior, affect, and meaning, and understanding the manifestations of trauma in each domain. Every session also included a check‐in that reviewed changes occurring in PTSD symptoms based on a symptom list created during the first sessions, as well as reviewing homework assignments such as tracking sensations in the body and self‐regulating in‐between sessions. The traumatic events, or traumatic story, was gradually introduced during Sessions 3 and 4, and more fully delved into during Sessions 5 through 11. The therapeutic work focused on using the traumatic story, or parts of it, to trigger low‐level autonomic nervous system activation, track bodily reactions and guide its discharge. In the final sessions, the work centered on how to maintain therapy successes, manage stress levels, and look at future directions in life in the aftermath of trauma.

### Measures

Symptoms of PTSD were evaluated by the Clinician‐Administered PTSD Scale (CAPS; Blake et al., 1995). The Hebrew version of the scale has been extensively used and validated (Shalev, Freedman, Peri, Brandes, & Sahar, 1997). The CAPS, a 30‐item structured interview, corresponds to the criteria for PTSD and is considered the gold standard in PTSD assessment (Weathers, Keane, & Davidson, 2001). The CAPS can be used to make a current (past month) or lifetime diagnosis of PTSD by diagnosing the 17 PTSD symptoms, and also gives a severity score. The CAPS was designed to be administered by clinicians and clinical researchers who have a working knowledge of PTSD, and can also be administered by appropriately trained paraprofessionals.

Exclusion criteria were assessed using the Hebrew version of the Structured Clinical Interview for *DSM‐IV* (Shalev, Abramovitz, & Kaplan‐De‐Nour, 1996). The SCID (Spitzer et al., 1992) is a semistructured interview that assesses 33 of the more commonly occurring psychiatric disorders described in the *Diagnostic and Statistical Manual of Mental Disorders* (4th ed., *DSM‐IV*; American Psychiatric Association, 1994). It allows the experienced clinician to tailor questions to fit the patient's understanding; to ask additional questions that clarify ambiguities; to challenge inconsistencies; and to make clinical judgments about the seriousness of symptoms. The main uses of the SCID are for diagnostic evaluation, research, and the training of mental health professionals. Information about the type of traumatic event and time passed since the event, were obtained from the clinical assessment.

The Posttraumatic Diagnostics Scale (PDS; Foa, Cashman, Jaycox, & Perry, 1997) was also used to assess posttraumatic stress symptoms. The Hebrew version was translated and validated (Foa, Doron, & Yadin, 2011). The PDS is a 49‐item self‐report measure for adults. It yields a total score (range = 0 to 51) that reflects the frequency of 17 symptoms of PTSD in the past month. Additionally, the PDS provides a count of the number of symptoms endorsed, a rating of symptom severity, and a rating of functional impairment. In the current study, a total severity score of posttraumatic symptoms that reflects the severity of posttraumatic distress was calculated as a continuous measure. The internal reliability of the severity score was measured by Cronbach's α (α = .85).

The participants' symptoms of depression were assessed by the Center for Epidemiological Studies Depression Scale (CES‐D; Radloff, 1977). The CES‐D is a 20‐item self‐report scale that measures symptoms of depression in general populations. Each item is rated on a 4‐point scale (0 = *rarely or none of the time [less than 1 day] during the past week*, 3 = *most or all of the time [5–7 days] during the past week*). The responses are summed to a total score, which can range from 0 to 60. Cronbach's α was used to calculate reliability in the present study (α = .73). The Hebrew version of the scale has been extensively used (Soskolne, Bonne, Denour, & Shalev, 1996).

### Data Analysis

All analyses were conducted with SPSS software (Version 20.0). Nonresponse rates of posttraumatic symptom severity measured by the CAPS were 10.0% (6 missing) at T2 and 21.7% (13 missing) at T3. The Little MCAR test (Little, 1988) showed that we cannot assume data are missing completely at random, χ^2^ = 26.03, *df* = 14, *p* = .026 and the chi‐square test showed statistically significant dependence between nonresponse and time, χ^2^ = 14.95, *df* = 2, *p* < .001. The nonresponse rate of posttraumatic symptom severity measured by PDS was 8.3% (5 missing) at T2 and 21.7% (13 missing) at T3. The chi‐square test showed statistically significant dependence between nonresponse and time, χ^2^ = 13.18, *df* = 2, *p* = .001. The nonresponse rate of posttraumatic symptom severity measured by PDS was 10.0% (6 missing) at T2 and 21.7% (13 missing) at T3. The chi‐square test showed statistically significant dependence between nonresponse and time, χ^2^ = 12.26, *df* = 2, *p* = .002. Following Enders (2011), we estimated the model for testing the treatment effect using the linear mixed models module of SPSS (Shek & Ma, 2011). In this way, the data were analyzed using maximum likelihood imputation (Peugh & Enders, 2005) appropriate for handling designs with substantial dropout rates (Salim, Mackinnon, Christensen, & Kathleen, 2008). The model was calculated using the unstructured covariance matrix. Condition and time entered the model as dummy variables; the first measurement (T1) and the intervention condition were the reference categories. To test if the results were sensitive to the specific way the missing data imputation was handled, we estimated the same model using the SPSS generalized estimating equation module with multiple imputation data and received similar results.

## Results

The effect of the SE treatment was estimated using linear mixed modeling with condition (intervention and waitlist) and time (T1, T2, and T3) as factors, and the severity of posttraumatic symptoms, measured by the CAPS and the PDS, and depression, measured by the CES‐D, as the dependent variables. Reference categories were T1 and waitlist control group. Table 2 presents the descriptive statistics resulting from linear mixed model regression analysis of the intervention group and the waitlist group based on estimated marginal means and standard errors of the fitted model. The mixed model shows that both at the first measurement of the two groups and at the third measurement, after both groups received treatment, the assessment of posttraumatic symptoms based on the CAPS showed no statistically significant differences (see Table 3, model's effects). At T1, no differences were found between the groups (*B* = 0.45, *t* = 0.09, *df* = 60, *p* = .929 for CAPS; *B* = 0.05, *t* = 0.02, *df* = 59.94, *p* = .982 for PDS; *B* = 1.88, *t* = 0.76, *df* = 59.79, *p* = .448 for CES‐D), and at T3, the difference between the groups was once again nonsignificant (*B* = −8.08, *t* = −1.24, *df* = 56.27, *p* = .221 for CAPS; *B* = −2.19, *t* = −0.70, *df* = 53.90, *p* = .484 for PDS; *B* = −3.58, *t* = −1.05, *df* = 52.28, *p* = .297 for CES‐D). At the T2 measurement, at which point the intervention group had received treatment and the waitlist group had not, the level of posttraumatic symptoms (based on the CAPS, PDS, and CES‐D) in the intervention group decreased significantly, whereas those of the waitlist group remained stable (*B* = −22.76, *t* = −3.62, *df* = 54.26, *p* = .001 for CAPS; *B* = −11.19, *t* = −4.06, *df* = 55.82, *p* < .001 for PDS; *B* = −10.68, *t* = −3.29, *df* = 55.92, *p* = .002 for CES‐D). To test the general decline in symptoms between T1 and T3, models with only main effects were calculated. Those models confirmed that the general decline in symptoms was statistically significant (*B* = −26.35, *t* = −7.95, *df* = 55.99, *p* < .001 for CAPS; *B* = −26.35, *t* = −11.01, *df* = 55.31, *p* < .001 for PDS; *B* = −10.14, *t* = −5.91, *df* = 52.08, *p* < .001 for CES‐D). On the clinical level, as measured by the CAPS, the diagnosis of PTSD was reversed for 44.1% of the sample through treatment, and this was maintained at follow‐up.

**Table 2:** Estimated Marginal Means and Standard Errors of Fitted Models by Mixed Model Regression Analysis

	T1		T2			T3		
Variables	*M*	*SE*	*M*	*SE*	Effect size T1–T2 (Cohen's *d*)	*M*	*SE*	Effect size T1–T3 (Cohen's *d*)
CAPS								
Intervention	68.37	3.49	36.31	5.30	1.26	37.53	5.08	1.25
Waitlist	67.93	3.73	58.62	5.40	0.38	45.16	5.33	0.94
PDS								
Intervention	34.13	1.60	21.36	2.18	1.18	21.69	2.22	1.13
Waitlist	34.08	1.73	32.50	2.19	0.15	23.83	2.32	0.95
CESD								
Intervention	37.26	1.67	24.14	2.52	1.08	25.21	2.52	1.00
Waitlist	35.38	1.81	32.94	2.49	0.21	26.91	2.68	0.70

*Note*. CAPS = Clinician‐Administered PTSD Scale; PDS = Posttraumatic Diagnostics Scale; CES‐D = Center for Epidemiological Studies Depression Scale.

**Table 3:** Mixed Model Predictors of Posttraumatic Symptom Severity and Depression

	CAPS				PDS				Depression			
Effect	*B*	*SE*	*df*	*t*	*B*	*SE*	*df*	*t*	*B*	*SE*	*df*	*t*
Intercept	67.93	3.66	60.00	18.54*	34.08	1.73	60.33	19.72*	35.38	1.81	60.16	19.55*
T1^a^												
T2	−9.31	4.45	54.13	−2.09*	−1.59	1.95	55.68	−0.81	−2.44	2.30	55.35	−1.06
T3	−22.77	4.72	58.31	−4.82*	−10.25	2.25	55.78	−4.56*	−8.47	2.49	54.83	−3.40**
Intervention	0.45	5.02	60.00	0.09	0.05	2.35	59.94	0.02	1.88	2.46	59.79	0.76
Control^a^												
T1^a^ × Intervention												
T1 × Control^a^												
T2 × Intervention	−22.76	6.29	54.26	−3.62*	−11.19	2.76	55.82	−4.06*	−10.68	3.29	55.92	−3.25**
T2 × Control^a^												
T3 × Intervention	−8.08	6.53	56.27	−1.24	−2.19	3.10	53.90	−0.70	−3.58	3.40	52.28	−1.05
T3 × Control^a^												
Information criteria												
Baseline model	1,413.84^b^		9		1,170.30		9		1,203.08^b^		9	
Full model	1,397.66^b^		12		1,147.15^b^		12		1,193.03^b^		12	
Δχ^2^	16.18^b^, *		3		23.15^b^, *		3		10.78^b^, **		3	

*Note*. CAPS = Clinician‐Administered PTSD Scale; PDS = Posttraumatic Diagnostics Scale; CES‐D = Center for Epidemiological Studies Depression Scale.

^a^Redundant. ^b^Value presented as 2 log likelihood.

*p < .05. **p < .01.

## Discussion

In this first randomized controlled study of SE for PTSD we have found that SE is an effective treatment for PTSD. The sample consisted of people who experienced a variety of trauma an average of four years before entering treatment; most trauma was civilian in nature, although some participants experienced combat or terrorist incidents. In meta‐analyses (e.g., Cusack et al., 2016) a number of trauma‐focused treatments have been found effective and insufficient evidence was found for differential effects between methods. In the number needed‐to‐treat analysis (Shearer‐Underhill & Marker, 2010) the results of this study fall in the range of the effective therapies (<4), meaning there is good reason to include SE in this category.

The results presented in this study show a large effect size (Cohen's *d* > 0.8) both on PTSD symptoms and depression symptoms, even though the clinical results should be considered moderate (44.1% lost the diagnosis of PTSD). The intervention was conducted during a period of ongoing collective trauma and unsafety due to political unrest in Israel, which included two wars (2012 and 2014) and ongoing terrorist attacks. Although it is difficult to assess the impact of these wars and ongoing violence on the scores of the participants, it is clear that the research took place in an ongoing traumatic field, for both participants and therapists. One of the most extreme examples of this is one participant who came in for treatment a few years after she was the victim of a terrorist attack. During the treatment and follow‐up period, two additional terrorist attacks took place in the neighborhood in which she lived. Another participant was the person in charge of attending to victims of terrorist attacks in his community for the last 20 years, and while in treatment was still holding the same job.

SE is a treatment modality that allows therapists a different therapeutic stance from other therapies, both by allowing healing without the full explicit retelling of the traumatic events, and by focusing on releasing bodily tensions in the therapeutic process. In some ways, SE does resemble mindfulness practices that have become part of many therapeutic approaches, as well as the focus on nervous system activity through neurofeedback (van der Kolk et al., 2016). The direction of attention in SE, however, is more on bodily sensations and the way they change. Attention is led to positive sensations first and only in a second phase to the balance between positive/pleasant sensations and negative/unpleasant sensations.

In light of the positive results of this study, we propose further studies look at SE effectiveness on more specific groups such as military trauma, sexual assault, and complex trauma. These different kinds of traumatic experiences each have their own characteristics in terms of the preponderance of hyperarousal in combat, intrusion of private space in sexual assault, and dissociative features in complex trauma. The state of the art in therapy outcome studies for PTSD seems to indicate that different therapies show similar results, and there are hardly indications that can be used to choose for one specific therapy. Knowing this, our next endeavor should be to look at how we can take care of all those who are not helped enough by the different methods. The study by Haagen et al. (2015) suggests that attention should be paid to those with high symptom severity levels. Between 30 and 60% of patients do not lose their diagnosis during therapy, even though their symptoms might go down significantly (e.g., Eftekhari, Ruzek, Crowley, Rosen, Greenbaum, & Karlin, 2013). Despite the relatively similar results of different modalities, not enough studies have been conducted to assess and compare differential effects. Also, more specific samples might give us a lead as to what SE might specifically be best for. Finally, there is a need to conduct process research on SE so that we might get a better handle on the curative mechanisms. Process research in the treatment of PTSD will need to involve physiological parameters. The theoretical foundation of SE provides leads for the measurement of physiological processes during the treatment.

There are some limitations in the present study. First, the study used a relatively small sample in a community setting. This is different from a sample derived from a university setting after predefined traumatic events. Second, although all therapists were closely supervised in their adherence to the treatment protocol, no measure of the behavioral adherence to the protocol was used. This is due to the dynamic nature of the process in SE that allows a limited form of strict protocol. In the first three to five sessions which focus on psychoeducation and the traumatic events, the protocol is quite clear and strict. From the fifth session, SE is practiced through the application of theoretical principles and their translation into observable phenomena. For this reason, in this phase it is difficult to prescribe the therapeutic behavior of the therapist. All of these limitations make this a naturalistic study that might be close to general clinical practice, but does not give us information about the comparison with other treatment modalities.
